# Chimney sweeps in Sweden: a questionnaire-based assessment of long-term changes in work conditions, and current eye and airway symptoms

**DOI:** 10.1007/s00420-016-1186-7

**Published:** 2016-11-17

**Authors:** Ayman Alhamdow, Per Gustavsson, Lars Rylander, Kristina Jakobsson, Håkan Tinnerberg, Karin Broberg

**Affiliations:** 1 Division of Occupational and Environmental Medicine, Department of Laboratory Medicine, Lund University, Lund, Sweden; 2 Unit of Metals and Health, Institute of Environmental Medicine, Karolinska Institutet, Nobels Väg 13, 171 77 Stockholm, Sweden

**Keywords:** Chimney sweeping, Cough, Occupational, Protection, Black soot

## Abstract

**Objectives:**

To explore chimney sweeping work tasks, chimney sweeps’ use of protective equipment, and type of fuel used by clients, over time. Further, to assess work-relatedness of current eye and airway symptoms.

**Methods:**

In a cross-sectional study in 2011, male Swedish chimney sweeps (*n* = 483; age 21–69 years) answered a questionnaire about their occupational history and eye and airway symptoms.

**Results:**

Between 1960 and 2010, black-soot-sweeping in private homes was the major task, although it decreased during the time period, for chimney sweeps. Between 1975 and 2010, the use of petroleum oil decreased, whereas the use of pellets and wood increased. Also, the use of gloves and masks increased significantly. Black-soot-sweeping in industry was associated with work-related eye symptoms (prevalence odds ratio POR = 3.76, 95% CI: 1.72–8.24, for every 10% increment of working time, adjusted for age and tobacco smoking). Chimney sweeps also had slightly higher prevalence of cough with increasing black-soot-sweeping (POR = 1.06, 95% CI: 0.99–1.13 for every 10% increment, further adjusted for the use of mask), and the association was more pronounced, although nonsignificant, for black-soot-sweeping in industry (adjusted POR = 1.26, 95% CI: 0.98–1.61).

**Conclusions:**

Chimney sweeping tasks and use of protective equipment as well as type of fuel used by the clients changed significantly over the last 35 years, which may have changed chimney sweeps’ exposure to soot. Still, chimney sweeps in Sweden have black-soot-sweeping-related eye and airway symptoms.

**Electronic supplementary material:**

The online version of this article (doi:10.1007/s00420-016-1186-7) contains supplementary material, which is available to authorized users.

## Introduction

The chimney sweeping profession basically involves two kinds of work, “black sweeping” and “white sweeping.” Black sweeping includes removal of black soot from oil-, wood-, and pellet-based boilers and wood-based stoves and fireplaces in private homes. In the industrial settings, black sweeping entails removing black soot from large boilers and associated channels. White sweeping includes inspection of fire-safety systems, boilers, and furnaces, as well as cleaning ventilation channels and exhaust ducts in restaurants, and performing mandatory ventilation inspection. Wood, wood pellets, coal, coke, oil, and natural gas are the most common materials used to produce energy or heat by combustion in Sweden (Andersson 1987). The content and quantity of the toxic compounds in black soot produced by combustion can differ considerably depending on the fuel (IARC 2012; Lighty et al. 2000). Furthermore, a Swedish report indicates that the type of fuel used in private homes and the industry has changed during the last 50 years, as oil has gradually been replaced by other types of fuels (AG-Framtid 2008). However, how this has influenced the exposure profile of chimney sweeps remains unknown. Furthermore, the awareness of safety at work may also have changed over time, but how this has affected the use of protective equipment has not been studied.

Chimney sweeps are exposed to harmful agents from black-soot-sweeping, such as carbon particles, multi-benzene-ring derivatives of polycyclic aromatic hydrocarbons (PAHs), metals and metalloids (e.g., nickel, arsenic, lead, chromium, and cadmium), and combustion gases (carbon monoxide, carbon dioxide, sulfur compounds, and nitrogen oxides) (Andersson 1987; IARC 2012; Lighty et al. 2000). Chimney sweeps are also exposed to organic solvents, detergents, asbestos, and dust during both black and white sweeping (Fehrmann 1982; IARC 2012; Sheehan et al. 2011). Exposure to these chemicals occurs by inhalation, ingestion, and dermal contact, which may cause various health problems. Chimney sweeps are at higher risk for cancer in the bladder, lung, esophagus, bowel, pleura, colon, and liver as well as hematopoietic cancers (Evanoff et al. 1993; Gustavsson et al. 1987; Hogstedt et al. 2013; Jansson et al. 2012). Moreover, increased incidence of myocardial infarction and mortality from cardiovascular and respiratory diseases has been reported (Gustavsson et al. 1987, 2013; Hansen 1983; Jansson et al. 2012). Chimney sweeps also have an increased risk of asthma (Li et al. 2008) and increased prevalence of long-term cough accompanied with phlegm, dyspnea, and substernal distress (Hansen 1990). However, symptoms from airways and eyes are not well characterized among chimney sweeps, and particularly not in relation to work tasks, despite the fact that many of the chimney sweeping-related compounds are airway and skin irritants.

The aims of this study were twofold: to explore long-term changes of Swedish chimney sweeps’ work tasks, the use of protective equipment and the use of fuel by clients, and further, to study eye and airway symptoms among chimney sweeps in relation to their work tasks.

## Methods

### Study group

In a cross-sectional design, chimney sweeps were recruited between February 2011 and June 2011 with two reminders in March and June. From the trade union we obtained the addresses of all chimney sweeps (*n* = 784) that were union members. We mailed the questionnaires to the union members and a total of 403 chimney sweeps answered and returned their questionnaires to the Division of Occupational and Environmental Medicine, Lund University. We enrolled additional 109 chimney sweeps that were not union members via direct contact with employer organizations. This type of recruitment was performed in order to enroll as many chimney sweeps as possible to the study. The total number of chimney sweeps that were not union members was unknown, and therefore, the exact participation rate of the study could not be calculated. However, based on communication with the union and the employer organization, we estimate that there are around 1500 chimney sweeps currently working in Sweden and with the total of 512 chimney sweeps that answered the questionnaires, the participation rate was around 30%. For the analysis, we excluded 15 females, due to large gender imbalance, and 14 males that returned the questionnaires without answering any question, which resulted in 483 as a final number of participants for further analysis.

### Questionnaire

Chimney sweeps were asked to estimate the percentage of time spent for each work task of chimney sweeping (i.e., black-soot-sweeping in private homes, black-soot-sweeping in industrial settings, inspection of fire-safety systems and boilers, cleaning ventilation channels, conducting mandatory ventilation inspection, cleaning exhaust ducts in restaurants, and office work) during the time period “the past 12 months,” and every 10-year interval back to 1960 for as long as they had been working as chimney sweeps. For the subsequent questions about the use of fuel by clients and work practices, only three periods were indicated (“the past 12 months,” “from 2000 to 2009,” and “from 1975 to 1999”) to limit the length of the questionnaire. The sweeps were asked to assess the use of fuel by clients by estimating the percentage of time spent cleaning oil-, wood-, and pellet-based boilers, and wood-based fireplaces (i.e., black-soot-sweeping), in private homes and industry. To note, not all chimney sweeps worked continuously during the whole time periods. Some sweeps may have worked from 1970 to 1979 and stopped during the next 10 or 20 years and then worked again during the subsequent periods.

Chimney sweeps should ideally use gloves, masks, long-sleeved shirts and pants, overall protectors and vacuum machines during work, especially during black sweeping. Nevertheless, the extent of use of each protective measure may considerably vary between different work tasks. We did not ask for the exact type of clothes, masks, and gloves that were in use; however, according to information from the trade union (personal communication), two types of masks were used: simple and advanced masks (Fig. 3). The chimney sweeps were asked to estimate the percentage of use of every protective measure separately for five different major work tasks (i.e., black-soot-sweeping in private homes, black-soot-sweeping in industry, inspection of fire-safety systems and boilers, cleaning ventilation channels, and cleaning exhaust ducts in restaurants) over three periods (“the past 12 months,” “from 2000 to 2009,” and “from 1975 to 1999”). Further questions were also asked regarding type of mask and frequency of changing gloves (“every day,” “once/week,” “once/month,” “rarely”) over the time periods.

We essentially focused on the following outcomes: eye symptoms (running, itching, stinging, burning), nose symptoms (running, itching, congestion, sneezing), nose bleeding, wheeze (whistling sound during breathing), and cough. The questions on these symptoms were mainly derived from a previously reported questionnaire used for other occupations (Littorin et al. 2007) and concerned the past 12 months of work (yes/no). For instance, one question asked “have you had eye symptoms during the past twelve months?” Furthermore, when participants answered “yes” on symptoms, there were three follow-up questions to identify work-related symptoms: “do the symptoms increase during work?”, “do the symptoms decrease on weekends?”, and “do the symptoms decrease after more than one week of rest?” For the statistical analysis we used the last question for assessment of work-related symptoms (Littorin et al. 2007). We also asked the chimney sweeps about other common diseases, i.e., asthma (“Do you have asthma diagnosed by a doctor?”), atopy (“Have you had, during childhood or juvenescence, atopic dermatitis, urticaria, hay fever or other kinds of allergic rhinitis?”), eczema (“Do you have or have you had hand eczema?”), and chronic lung/heart disease (“Do you have any chronic lung or heart disease?”).

Chimney sweeps were further asked questions regarding tobacco smoking (“daily smoker,” “occasionally smoker,” “ever smoker,” and “never smoker”) and alcohol consumption (“≤once/month,” “2–4 times/month,” “2–3 times/week,” and “≥4 times/week”); each drinking occasion includes at least 50 ml regular beer (≤3.5 vol%), 33 ml strong beer (>3.5 vol%), 10–15 ml white or red wine, 5–8 ml fortified wine, or 4 ml liquor (e.g., whiskey).

### Statistical analysis

The questionnaire covered the periods from 1975 to 1999, from 2000 to 2009, and past 12 months; we set the year 2000 as an arbitrary cutoff to evaluate changes in use of fuel, work tasks and use of protective measures before and after this year. Thereafter, Wilcoxon signed-rank tests were performed to explore changes for chimney sweeps’ work tasks, chimney sweeps’ use of protective clothing and equipment, and the use of fuel by clients. Logistic regression analysis was used to estimate prevalence odds ratio (POR) of having symptoms or disease in relation to different work tasks during the past 12 months. The POR estimated the risk of a specific symptom with a 10% increase of the work task. We focused primarily on black-soot-sweeping in private homes and industrial settings, since black soot particles have been associated with airway diseases (Barraza-Villarreal et al. 2014; Nwokoro et al. 2012; Suresh et al. 2009). Potential confounders (age, smoking, and use of mask) were included in the analysis due to their potential role in developing or preventing symptoms of interest (Supplementary Table 6). However, we excluded the use of mask in the models for eye symptoms, due to irrelevancy. We had two categories for tobacco smoking: non-smokers (never smokers) and smokers (daily, occasionally, and ever smokers). We did not include atopy, asthma, or chronic lung/heart disease in the analyses since they were not associated with work tasks as evaluated by univariate logistic regression analysis. Spearman’s rank correlations were employed to investigate correlations between non-dichotomous variables, whereas Fisher’s exact test was used for investigating associations between categorical variables, i.e., tobacco smoking (non-smoker/smoker) and symptoms (yes/no).

All the statistical analyses were performed with SPSS software version 21.0 (SPSS Inc, Chicago, IL, USA), and statistical significance refers to *p* < 0.05.

## Results

### General characteristics of the chimney sweeps

Table 1 shows the general characteristics of the chimney sweeps in this study. The chimney sweeps were from different parts of Sweden; about half of them were from southern Sweden, and the majority of the sweeps lived in small cities. One-fourth of the participants had been working as chimney sweeps for >31 years and only 6% for ≤1 year. The frequency of regular alcohol drinkers (≥4 times/week) was 1.2%, and more than half of the sweeps were non-smokers.

**Table 1:** Characteristics of chimney sweeps assessed at the time of study participation

Variable	Total number of respondents	*N*	%^a^
Age, median years (range)	483	46 (21–69)	100.0
Smoking habits	480		
Non-smoker		279	57.8
Smoker		201	41.6
Alcohol consumption (yes)	479		
≥4 times/week		6	1.2
2–3 times/week		87	18.0
2–4 times/month		247	51.1
≤once/month		139	28.8
Place of residence (city)	482		
Small city (<100,000 inhabitants)		437	90.5
Medium city (100,000–300,000 inhabitants)		24	5.0
Big city (>300,000 inhabitants)		21	4.3
Place of residence (province)	482		
Götaland (southern Sweden)		220	45.5
Svealand (middle Sweden)		156	32.3
Norrland (northern Sweden)		106	21.9
Working years	474		
≤1		27	5.6
>1–11		113	23.4
>11–21		105	21.7
>21–31		111	23.0
>31–41		70	14.5
>41–51		48	9.9
Atopy (yes)	475	102	21.1
Chronic lung/heart disease (yes)	458	22	4.6
Asthma (yes)	472	38	7.9

^a^Calculated based on the total number of participants (*n* = 483)

### Work tasks and the use of fuel over time

**Fig. 1:**
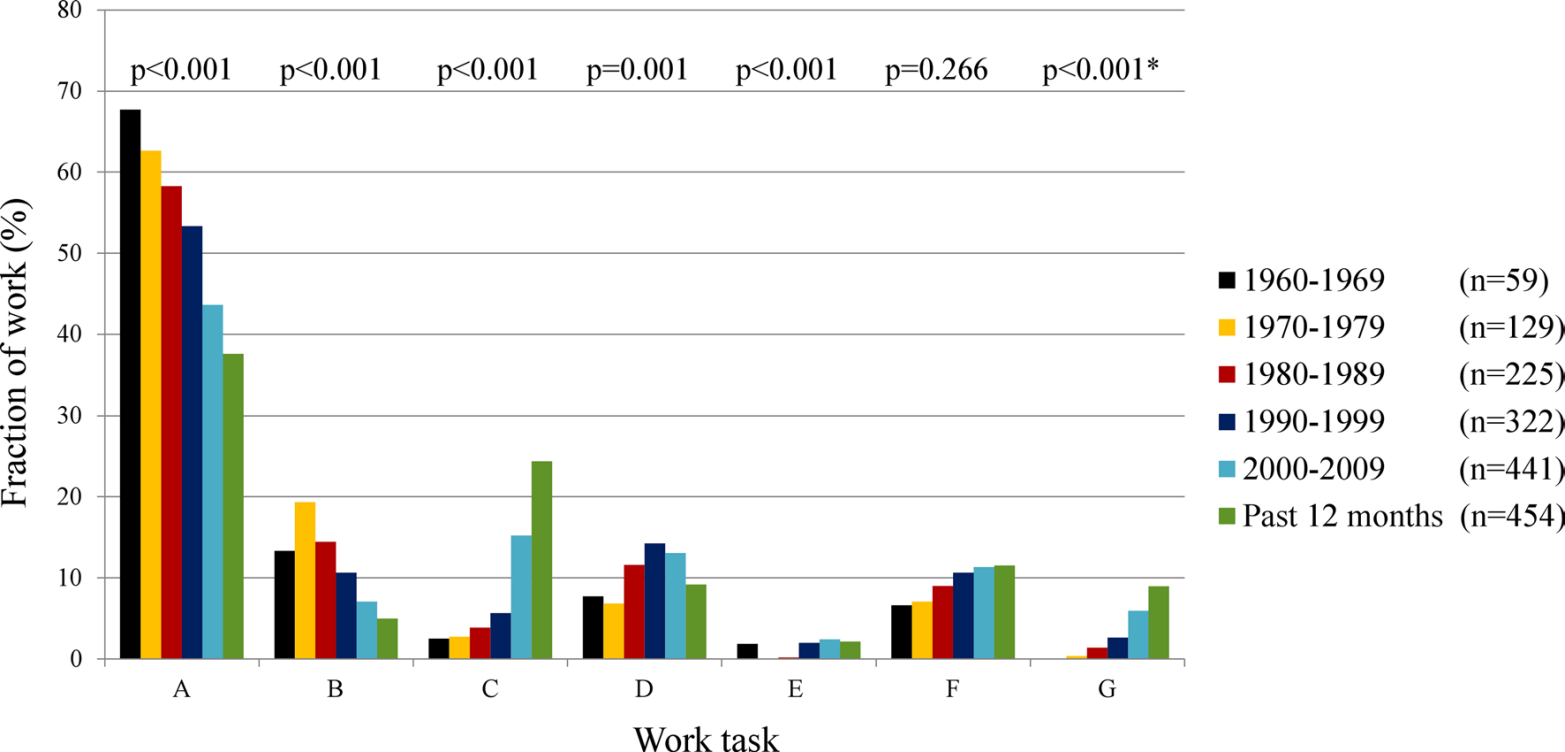
Bar chart of fraction of work (%) with different work tasks from 1960 to 2010. *Wilcoxon signed-rank test for work tasks before/after the year of 2000. **a** Black-soot-sweeping in private homes. **b** Black-soot-sweeping in industry. **c** Inspection of fire-safety systems, boilers, and furnaces inspection. **d** Cleaning ventilation channels in houses, buildings, and industry. **e** Mandatory ventilation inspection. **f** Cleaning exhaust ducts in restaurants. **g** Office work

The major task for chimney sweeps from 1960 to 2010 was, by far, black-soot-sweeping in private homes (Fig. 1). Comparing the percentages of work before and after 2000, chimney sweeps worked after 2000 significantly less with black-soot-sweeping in private homes and the industry, whereas they worked significantly more with inspection of fire-safety systems, mandatory ventilation inspection, and office work (*p* ≤ 0.001 for all tasks). Younger sweeps were to a higher extent responsible for tough/dirty tasks such as black-soot-sweeping, compared with senior sweeps (Supplementary Table 1).

**Fig. 2:**
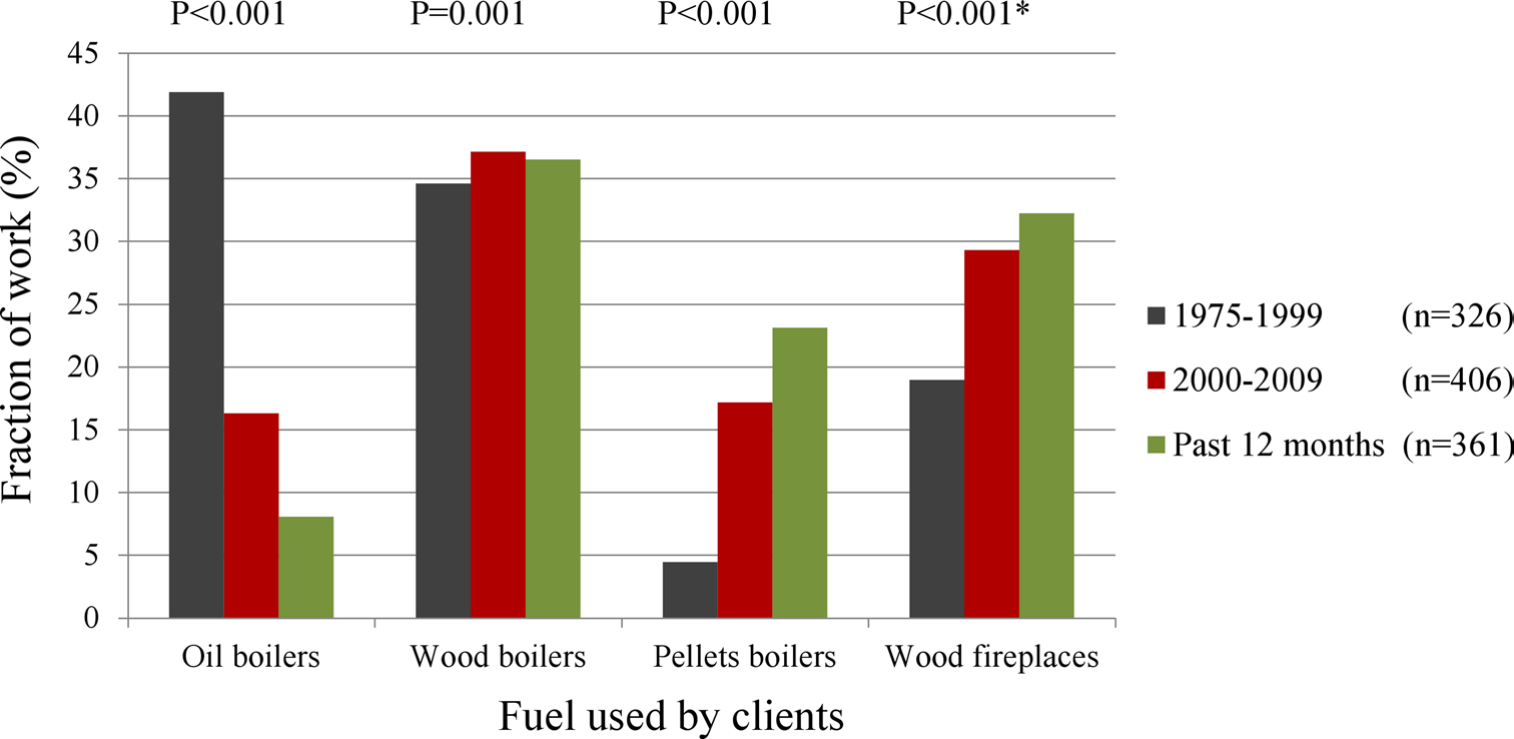
Fraction of work (%) with different kinds of fuels used by clients from 1975 to 2010. *Wilcoxon signed rank test for the use of fuel by clients before/ after the year of 2000

The use of fuel by the clients also changed from 1975 to 2010 (Fig. 2). After 2000, the use of petroleum oil decreased (*p* < 0.001), while the use of pellets and wood increased (*p* ≤ 0.001).

### Use of protection

#### Gloves

The use of gloves increased significantly from 1975 to 2010 for all work tasks (Table 2); however, the increase was not dramatic for black-soot-sweeping in private homes and industry; the mean of use of gloves slightly increased from 80.6% in 1975–1999 to 84.1% in the past 12 months when working with black-soot-sweeping in private homes, and from 85.5% in 1975–1999 to 86.9% in the past 12 months for black-soot-sweeping in industry. Further, approximately half of the chimney sweeps reported changing gloves “once a week” and about 40% reported “rarely” during the entire time period (Supplementary Table 2). The percentage of use of gloves was approximately twice as high when doing black-soot-sweeping in private homes and industry compared to other tasks. In general, younger chimney sweeps used gloves more often compared to older sweeps (Supplementary Table 3).

**Table 2:** Use of protective clothing and equipment by chimney sweeps for different work tasks 1975–2010 (mean percentage)

	*N*	BS^a^ homes	*N*	BS^b^ industry	*N*	Fire-safety^c^	*N*	Ventilation^d^	*N*	Exhaust ducts^e^
Gloves										
1975–1999	325	80.6	324	85.5	184	40.2	290	43.1	293	40.2
2000–2009	426	84.5	413	88.2	301	50.1	411	51.3	411	52.8
Past 12 months	418	84.1	404	86.9	318	53.1	394	51.7	405	54.4
*P* value^f^		<0.001		0.023		<0.001		<0.001		<0.001
Masks										
1975–1999	379	14.9	329	48.6	80	5.1	197	20.3	109	4.6
2000–2009	435	19.9	418	57.5	141	11.7	269	25.8	165	9.3
Past 12 months	431	20.2	392	57.7	143	14.5	261	24.7	171	11.5
*P* value		<0.001		<0.001		0.001		<0.001		<0.001
Long sleeves										
1975–1999	317	87.5	310	93.4	194	76.9	295	77.9	307	78.2
2000–2009	420	83.2	407	90.5	294	72.5	404	74.6	411	75.3
Past 12 months	401	82.9	388	89.6	301	71.6	383	73.4	402	75.7
*P* value		0.068		0.778		0.593		0.132		0.281
Long pants										
1975–1999	316	98.1	311	98.0	202	89.5	299	96.6	306	97.7
2000–2009	418	96.7	407	96.4	294	86.9	407	96.2	409	96.8
Past 12 months	401	96.3	390	95.1	301	86.6	386	95.0	403	96.1
*P* value		0.010		0.148		0.710		0.650		0.620
Overall										
1975–1999	284	2.1	294	20.8	213	0.7	276	4.7	275	4.4
2000–2009	380	1.1	384	20.8	288	0.7	378	4.6	373	4.4
Past 12 months	381	1.3	380	19.2	295	0.8	374	3.9	379	3.9
*P* value		0.001		0.002		0.929		0.722		0.649
Vacuum machine										
1975–1999	317	55.4	308	51.7		–		–		–
2000–2009	419	68.4	403	60.8		–		–		–
Past 12 months	400	71.7	384	65.9		–		–		–
*P* value		<0.001		<0.001		–		–		–

^a^Black-soot-sweeping in private homes

^b^Black-soot-sweeping in industry

^c^Inspection of fire-safety systems, boilers, and furnace

^d^Cleaning ventilation channels in houses, buildings, and industry

^e^Cleaning exhaust ducts in restaurants

^f^ *P* value of Wilcoxon signed-rank test before/after the year of 2000

#### Masks

**Fig. 3:**
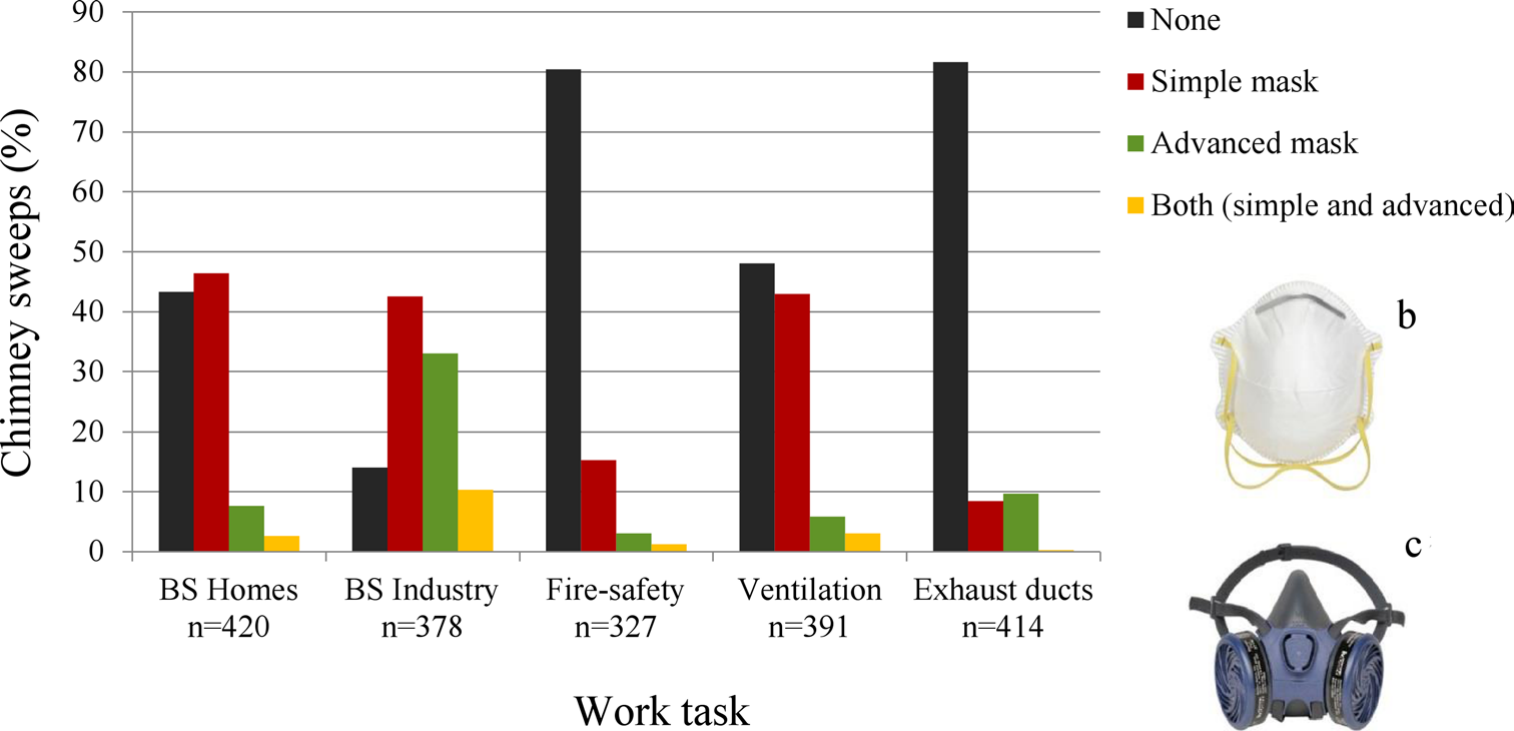
Bar chart showing the percentage of chimney sweeps that used mask during work for five different work tasks. **b** Simple mask. **c** Advanced mask

The use of masks increased significantly over time for all tasks (Table 2). More chimney sweeps used simple masks when cleaning ventilation channels and black-soot-sweeping in private homes and industry (Fig. 3). The advanced masks were to a much higher extent applied when working with black-soot-sweeping in industry compared to other tasks. However, about 80% of the sweeps did not use any mask during inspection of fire-safety systems and cleaning exhaust ducts. Younger sweeps employed masks more than older sweeps for all tasks (Supplementary Table 4) and over the whole time period (1975–2010) (Supplementary Table 5).

#### Long sleeves and pants

The use of long sleeves and long pants slightly decreased over time for all tasks (Table 2). However, long pants were extensively employed in all tasks, particularly black-soot-sweeping in private homes and industrial settings (mean percentage of use ≥95%), apart from inspection of fire-safety systems, over the whole period.

#### Overall protectors

Overall protectors were rarely used, except for black-soot-sweeping in industry (the mean percentage of use for sweep in industry was around 20% over the three periods) (Table 2). No significant differences were seen in the use of overall protectors before and after the year 2000 for any work task.

#### Vacuum machine

Vacuum machine was used during black-soot-sweeping in private homes and industry (Table 2). The use of this device increased significantly after the year of 2000.

### Symptoms

We found that the prevalence of cough increased among chimney sweeps with the increasing proportion of time spent doing total, i.e., in private homes and industry, black-soot-sweeping (for every 10% increment, crude prevalence odds ratio, POR = 1.05, 95% CI: 1.00–1.11). This association was nonsignificant after adjustment for age, smoking, and use of mask POR = 1.06; 95% CI: 0.99–1.13 (Table 3a). Work-related symptoms showed higher but nonsignificant effect estimates. When analyzing symptoms in association with black-soot-sweeping in private homes and industry separately (Table 3b, c), we found statistically significant increased prevalence of cough and work-related eye symptoms for increasing black-soot-sweeping in the industry. However, the prevalence of cough was nonsignificant in the adjusted analysis (*p* = 0.074). Work-related nasal symptoms showed higher, but nonsignificant estimates with increasing black-soot-sweeping in industry (*p* ≤ 0.066, for both crude and adjusted models). The use of masks had a protective effect against total/work-related symptoms, apart from work-related nasal symptoms, in relation to black-soot-sweeping in the industry (data not shown).

**Table 3:** Logistic regression analysis of risk of symptoms for chimney sweeps (*n* = 483) in relation to work with black-soot-sweeping in (a) both private homes and industrial settings, (b) private homes, (c) industrial settings during the past 12 months

Symptoms	Total								Work-related							
	Crude				Adjusted^a^				Crude				Adjusted			
	*N*	OR	95% CI		*N*	OR	95% CI		*N*	OR	95% CI		*N*	OR	95% CI	
(a) Total black-soot-sweeping (in private homes and industry)																
Wheeze^b^	476	0.97	0.89	1.06	381	0.95	0.86	1.05	69	1.07	0.94	1.21	55	1.01	0.86	1.18
Cough^b^	481	1.05	1.00	1.11	384	1.06	0.99	1.13	143	1.08	0.97	1.19	117	1.08	0.95	1.23
Nasal symptoms^bc^	480	1.02	0.97	1.07	383	1.02	0.96	1.08	208	1.06	0.98	1.14	169	1.06	0.97	1.16
Eye symptoms^d^	478	1.00	0.95	1.05	476	0.99	0.94	1.05	133	1.06	0.97	1.17	132	1.07	0.97	1.17
Nasal bleeding	469	0.99	0.93	1.05	375	0.96	0.89	1.04	78	1.11	0.98	1.26	68	1.08	0.94	1.24
(b) Black-soot-sweeping in private homes																
Wheeze^b^	449	0.97	0.88	1.06	413	0.94	0.85	1.04	63	1.03	0.89	1.20	59	1.02	0.87	1.20
Cough^b^	453	1.04	0.98	1.10	416	1.03	0.97	1.10	136	1.03	0.92	1.16	128	1.03	0.91	1.17
Nasal symptoms^bc^	452	1.01	0.96	1.07	415	1.00	0.94	1.06	197	1.05	0.97	1.14	183	1.05	0.97	1.15
Eye symptoms^d^	450	0.99	0.94	1.06	449	0.99	0.93	1.05	124	1.04	0.93	1.15	124	1.03	0.93	1.15
Nasal bleeding	442	0.97	0.90	1.04	405	0.94	0.88	1.02	76	1.11	0.97	1.27	73	1.06	0.92	1.23
(c) Black-soot-sweeping in the industry																
Wheeze^b^	449	1.21	0.93	1.59	376	1.11	0.83	1.47	63	1.26	0.80	1.98	55	1.23	0.76	1.98
Cough^b^	453	1.26	1.01	1.58	379	1.26	0.98	1.61	136	0.93	0.69	1.27	116	1.09	0.75	1.60
Nasal symptoms^bc^	452	1.07	0.87	1.32	378	1.08	0.87	1.34	197	1.59	0.99	2.56	167	1.66	0.97	2.85
Eye symptoms^d^	450	1.11	0.89	1.39	449	1.12	0.90	1.40	124	3.86	1.77	8.45	124	3.76	1.72	8.24
Nasal bleeding	442	1.03	0.80	1.33	370	1.02	0.77	1.35	76	1.19	0.76	1.86	67	1.25	0.77	2.01

Effects are presented as odds ratio (OR) per 10% increment of fraction of work hours, in crude and adjusted models [95% CI = 95% confidence interval]

^a^Age, smoking status, and mask use (except for eye symptoms) were considered for adjustment

^b^Without having a cold

^c^Congestion, sneezing, itchy, or runny nose

^d^Burning, stinging, runny, or itchy eye

The prevalence of cough decreased with the increasing percentage of time spent on mandatory ventilation inspection (adjusted POR = 0.64; 95% CI: 0.42–0.97) (Supplementary Table 7c). Similarly, the prevalence of work-related eye (adjusted POR = 0.73; 95% CI: 0.54–0.98) and nasal symptoms (adjusted POR = 0.73; 95% CI: 0.57–0.93) decreased with the increasing percentage of time spent on office work (Supplementary Table 7e). Also, a decreased prevalence of eye symptoms was seen with increasing inspection of fire-safety systems (adjusted POR = 0.85; 95% CI: 0.75–0.96) (Supplementary Table 7a). Cleaning ventilation channels did not have any effect on symptoms (Supplementary Table 7b).

We also evaluated how years of employment influenced the prevalence of symptoms (data not shown) but found no significant associations.

## Discussion

Our results show that, over a period of 35 years, the work tasks of Swedish chimney sweeps have significantly changed, with decreasing work with traditional black-soot-sweeping and increasing work with other tasks, such as inspection of fire-safety systems and cleaning exhaust ducts. Furthermore, the use of protective clothing and equipment has significantly changed over time, with increasing use of gloves, masks, and vacuum machines, and decreasing use of clothes covering the arms and legs. Also, the type of fuel has changed from oil to pellets and wood. One can speculate that with these changes, the degree and type of exposure to toxic substances from chimney sweeping differ substantially today, compared to 35 years ago. Moreover, although black-soot-sweeping (the major suspected source of toxicants) still predominated, the reduction of time for this work task probably results in a change of the risk of diseases previously linked to chimney sweeping, i.e., cancer and cardiovascular diseases (Gustavsson et al. 2013; Hogstedt et al. 2013; Jansson et al. 2012).

Our results also show that chimney sweeps working with black-soot-sweeping in the industry today experience cough and work-related eye symptoms. In line with this finding, we observed a lower prevalence for eye and nasal symptoms with more office work. We also evaluated the impact of years of employment on the degree of symptoms, but found no significant associations, partly explained by the fact that some symptoms, such as eye and nasal symptoms, are more likely associated with short-term than with long-term exposure. Eye and airway symptoms among chimney sweeps have previously not been well studied, and our findings demonstrate that further precautionary measures need to be taken in this profession. In particular, the use of overall protectors, masks, and protective glasses should be encouraged and also alternative ways of performing black-soot-sweeping in the industry should be explored.

There are some methodological issues to consider with our study. The strengths were that it contained extensive details on chimney sweeping profession for a large group of currently working chimney sweeps and covered a time period over the last 35 years. However, we estimate that a significant number of chimney sweeps did not participate in our study and the relatively low participation rate might have biased the findings. Also, the retrospectively collected data in our study, especially the estimation of time percentage for each task and the extent of applying different protective measures during different time points, were hard to validate due to potential recall bias. It is therefore possible that we would have obtained different results for work tasks, use of fuel, and protective measures from the same chimney sweeps if they were surveyed 20–30 years ago. Ostensibly, we had varying number of missing information for some variables related to work tasks, fuel used by clients, and protective clothing and equipment over multiple time periods. This is due to the fact that not all chimney sweeps in the study worked during all time periods or performed all work tasks. The exposure assessment was crude, and no biological samples or workplace air measurements were available. Instead, we created a measure of 10% increments of work fraction for each sweeping task. From the questionnaire data, some work-related symptoms (e.g., nasal bleeding) had low statistical power because we found few individuals with these symptoms. Moreover, performing multiple comparisons may have led to chance outcomes, and the health effects associated with chimney sweeping need therefore to be cautiously interpreted. We focused on airway and eye symptoms, which have not been well studied earlier; however, it would have been advantageous to have a matched control group for comparison of the prevalence of these symptoms among chimney sweeps.

Personal protection during chimney sweeping work is not mandatory; therefore, the sweeps wear and use what they think is necessary and convenient in order to do a particular task. Long shirts and pants were the most common dress but T-shirts and shorts were also worn, particularly during white sweeping. Our results show that chimney sweeps did not employ protective equipment and procedures properly during work. About 40 and 50% of the chimney sweeps reported that they did not use masks during black-soot-sweeping in private homes and ventilation cleaning, respectively, tasks that involve exposure to black soot and dust particles. The frequency of changing gloves was also low and did not differ much over the whole period of 35 years. About 50 and 40% of the sweeps changed gloves once a week and once a month, respectively. We do not know what kind of gloves they were using, but we speculate that they were using multipurpose gloves made of leather or some other special materials.

The chimney sweeping profession has a low job turnover rate, as it usually runs in families, and it is therefore not surprising to find that about half of the sweeps worked for more than 21 years. The type of tasks that the chimney sweeps are responsible for differed depending on their age. Younger sweeps were usually responsible for tough/dirty tasks such as black-soot-sweeping, whereas the senior ones were more responsible for tasks such as inspection of fire-safety systems and office work. This could explain why younger sweeps showed higher use of protective measures, masks in particular, and may reflect a higher self-awareness about health problems involved in this occupation.

We found higher prevalence of cough with more total black-soot-sweeping and black-soot-sweeping in the industry. This is notable as increased mortality from non-malignant lung disease has been reported among chimney sweeps (Jansson et al. 2012). However, it needs to be cautiously interpreted since the adjusted models for cough and work-related cough were not statistically significant. We also found a lower prevalence of cough with more mandatory ventilation inspection, which was expected because mandatory ventilation inspection is a clean work task. The frequencies of asthma, and chronic lung or heart disease among the chimney sweeps were relatively similar to the frequencies in the general adult population of Sweden (Bjerg et al. 2015; Montnemery et al. 1998).

We do not know what component of soot that causes the symptoms resulting from black-soot-sweeping in industrial settings. Soot consists of total carbon (up to 60%), inorganic, and organic compounds. The organic part mainly consists of PAHs (Watson and Valberg 2001). Depending on the type of fuel used, several PAHs have been detected in the breathing zone of chimney sweeps during black-soot-sweeping in systems that use oil and solid fuel (Knecht et al. 1989). Other studies have found high levels of inhalable combustion particulates (≥2.5 mg/m^3^) (Gustavsson et al. 2001) and dust (3–19 mg/m^3^) (Andersson 1987) during chimney sweeping. Moreover, supporting our findings for more symptoms in relation to work in the industry, unpublished data showed that the median of inhalable dust levels was 3.8 mg/m^3^ over 8-h sweeping in private homes, but it exceeded 1000 mg/m^3^ during sweeping in industrial settings (Tinnerberg H, personal communication). The occupational exposure to PAHs has also been correlated with non-malignant lung, eye, and skin health problems, in addition to cancer (ATSDR 2009). Workers in the rubber industry with exposure to inhalable PAHs demonstrated increased symptoms affecting the eye (Jonsson et al. 2007) and the airways (ATSDR 1995; Zuskin et al. 1996). A chamber study showed that wood smoke, at low levels of exposure, affected the airway tracts (Stockfelt et al. 2012). Taken together, these observations indicate that chimney sweeps are exposed to multiple harmful agents as they do black-soot-sweeping in a wide range of industrial plants with different exposure profiles. It is therefore hard to estimate the contribution of each toxicant to the adverse health effects of the chimney sweeps of our study, but dust and PAHs likely have important roles. However, airway symptoms could be induced not only from inhalation, but also from direct dermal contact with toxicants leading to systematic inflammation. Pyrene and benzo(a)pyrene, two PAHs in black soot, can be quantified by a tape-stripping method applied on different exposure sites on the chimney sweeps’ skin indicating dermal exposure to black soot (Kammer et al. 2011). Chimney sweeps can also be exposed to soot by direct contact with contaminated clothes, tools, and cars, or through oral uptake from contaminated foodstuff, while resting or driving.

The smoking habits of chimney sweeps today are similar to the general population (2012 figures) (Statistics Sweden 2014); 13% of the chimney sweeps reported current smoking, compared with 14% of the general population. This means that smoking has decreased substantially in this profession, as data from a 1972 national survey of Swedish chimney sweeps found that 67% of chimney sweeps were smokers (Swensson and Swensson 1972). We therefore do not think that smoking confounded the results of increased airway symptoms among chimney sweeps. It should be noted that the alcohol consumption of the chimney sweeps appeared not to be extreme; however, we could not evaluate the data in relation to the general population of Sweden.

To conclude, chimney sweeping tasks, chimney sweeps’ use of protective equipment, as well as type of fuel used by the clients changed significantly over the last 35 years. This might have changed chimney sweeps’ exposure to soot and other harmful substances. Nonetheless, Swedish chimney sweeps have black sweeping-related airway and eye symptoms and further studies are needed to characterize the current exposure of chimney sweeps.

## Electronic supplementary material

Below is the link to the electronic supplementary material.

Supplementary material 1 (DOCX 84 kb)
