# Supplementary material for: Chimney sweeps in Sweden: a questionnaire-based assessment of long-term changes in work conditions, and current eye and airway symptoms
Source: Int Arch Occup Environ Health. 2016 Nov 17;90(2):207–16. doi: 10.1007/s00420-016-1186-7 (PMC5263190; doi:10.1007/s00420-016-1186-7)
Supplement: Supplementary file 1 — Supplementary material 1 (DOCX 84 kb) [file 420_2016_1186_MOESM1_ESM.docx]

**Chimney sweeps in Sweden: a questionnaire-based assessment of long-term changes in work conditions, and current eye and airway symptoms**

Running head: Chimney sweeping practices and eye and airway symptoms

Ayman Alhamdow, BPharm^2*^, Per Gustavsson, MD, PhD^2^, Lars Rylander, PhD^1^, Kristina Jakobsson, MD, PhD^1^, Håkan Tinnerberg, PhD^1^, Karin Broberg, PhD^1,2^

^1^Division of Occupational and Environmental Medicine, Department of Laboratory Medicine, Lund University, Lund, Sweden

^2^Institute of Environmental Medicine, Karolinska Institutet, Stockholm, Sweden

*Correspondence to: Ayman Alhamdow

Postal address: Unit of Metals & Health, Institute of Environmental Medicine, Karolinska Institutet, Nobels Väg 13, Stockholm 171 77, Sweden

E-mail: [ayman.alhamdow@ki.se](mailto:ayman.alhamdow@ki.se)

Phone: +46735791789

**SUPPLEMENTARY MATERIAL**

**Supplementary table 1.** Spearman’s correlations for age and different work tasks 2000-2010 [r_s_= r squared for Spearman’s correlation]

|  |  | 2000-2009 | Past 12 months |
| --- | --- | --- | --- |
| BS Homes^a^ | r_s_ | -0.14 | -0.10 |
|  | p value | <0.01 | 0.03 |
|  | N | 441 | 454 |
| BS Industry^b^ | r_s_ | -0.06 | -0.10 |
|  | p value | 0.18 | 0.04 |
|  | N | 441 | 454 |
| Fire-safety^c^ | r_s_ | 0.03 | -0.01 |
|  | p value | 0.52 | 0.76 |
|  | N | 441 | 454 |
| Ventilation^d^ | r_s_ | -0.03 | -0.02 |
|  | p value | 0.58 | 0.60 |
|  | N | 441 | 454 |
| OVI^e^ | r_s_ | -0.01 | -0.05 |
|  | p value | 0.83 | 0.25 |
|  | N | 441 | 454 |
| Exhaust ducts^f^ | r_s_ | -0.12 | -0.13 |
|  | p value | 0.01 | <0.01 |
|  | N | 441 | 454 |
| Office^g^ | r_s_ | 0.10 | 0.05 |
|  | p value | 0.04 | 0.33 |
|  | N | 441 | 454 |

^a^Black-soot-sweeping in private homes

^b^Black-soot-sweeping in industry

^c^Inspection of fire-safety systems, boilers, and furnace inspections

^d^Cleaning ventilation channels in houses, buildings and industry

^e^Mandatory ventilation inspection

^f^Cleaning exhaust ducts in restaurants

^g^Office work

**Supplementary table 2.** Frequency of changing gloves 1975-2010

|  | 1975-1999 | | 2000-2009 | | Past 12 months | |
| --- | --- | --- | --- | --- | --- | --- |
|  | N | % | N | % | N | % |
| Every day | 11 | 3.4 | 23 | 5.4 | 30 | 6.7 |
| Once/week | 165 | 51.2 | 234 | 54.7 | 236 | 53.1 |
| Rarely | 145 | 45.1 | 170 | 39.7 | 177 | 39.8 |
| Never | 1 | 0.3 | 1 | 0.2 | 2 | 0.4 |
| Total number of respondents | 322 | 100.0 | 428 | 100.0 | 445 | 100.0 |

**Supplementary table 3.** Spearman’s correlations for age and the use of gloves from 1975 to 2010 [r_s_= r squared for Spearman’s correlation]

|  |  | 1975-1999 | 2000-2009 | Past 12 months |
| --- | --- | --- | --- | --- |
| BS Homes | r_s_ | -0.13 | 0.02 | 0.05 |
|  | p value | 0.02 | 0.67 | 0.31 |
|  | N | 325 | 426 | 418 |
| BS Industry | r_s_ | -0.10 | -0.01 | 0.02 |
|  | p value | 0.06 | 0.76 | 0.62 |
|  | N | 324 | 413 | 404 |
| Fire-safety | r_s_ | -0.27 | -0.11 | -0.04 |
|  | p value | <0.01 | 0.06 | 0.50 |
|  | N | 184 | 301 | 318 |
| Ventilation | r_s_ | -0.15 | -0.07 | -0.03 |
|  | p value | <0.01 | 0.17 | 0.51 |
|  | N | 290 | 411 | 394 |
| Exhaust ducts | r_s_ | -0.15 | -0.15 | -0.09 |
|  | p value | 0.01 | <0.01 | 0.07 |
|  | N | 293 | 411 | 405 |

**Supplementary table 4.** Spearman’s correlations for age and the use of mask (yes/no) in different work tasks [r_s_= r squared for Spearman’s correlation]

|  |  | BS homes | BS industry | Fire-safety | Ventilation | Exhaust ducts |
| --- | --- | --- | --- | --- | --- | --- |
| Age | r_s_ | -0.12 | -0.10 | -0.14 | -0.12 | -0.13 |
|  | p value | 0.01 | 0.04 | 0.01 | 0.02 | <0.01 |
|  | N | 420 | 378 | 327 | 391 | 414 |

**Supplementary table 5.** Spearman’s correlations for age with the fraction of use of masks (%) during work from 1975 to 2010 [r_s_= r squared for Spearman’s correlation]

|  |  | 1975-1999 | 2000-2009 | Past 12 months |
| --- | --- | --- | --- | --- |
| BS homes | r_s_ | 0.04 | -0.05 | -0.10 |
|  | p value | 0.47 | 0.34 | 0.04 |
|  | N | 379 | 435 | 431 |
| BS industry | r_s_ | -0.15 | -0.09 | -0.10 |
|  | p value | <0.01 | 0.06 | 0.04 |
|  | N | 329 | 410 | 392 |
| Fire-safety | r_s_ | -0.10 | -0.22 | -0.23 |
|  | p value | 0.36 | <0.01 | <0.01 |
|  | N | 80 | 141 | 143 |
| Ventilation | r_s_ | -0.14 | 0.002 | -0.01 |
|  | p value | 0.05 | 0.97 | 0.81 |
|  | N | 197 | 269 | 261 |
| Exhaust ducts | r_s_ | -0.03 | -0.14 | -0.04 |
|  | p value | 0.76 | 0.07 | 0.56 |
|  | N | 109 | 165 | 171 |

**Supplementary table 6** Fisher’s Exact Test and Spearman’s correlations for potentially influential factors (smoking status and age) with total/work-related symptoms [r_s_= r squared for Spearman’s correlation]

| Symptoms | Total | | | Work-related | | |
| --- | --- | --- | --- | --- | --- | --- |
|  | Smoking status | Age | | Smoking status | Age | |
|  | p value^a^ | r_s_ | p value^b^ | p value | r_s_ | p value |
| Wheeze | <0.01 | 0.11 | 0.01 | 0.80 | 0.06 | 0.62 |
| Cough | <0.01 | -0.03 | 0.54 | 0.56 | -0.26 | <0.01 |
| Nasal symptoms | 0.92 | -0.15 | <0.01 | <0.01 | 0.02 | 0.75 |
| Eye symptoms | 1.00 | -0.11 | 0.01 | 0.38 | 0.02 | 0.85 |
| Nasal bleeding | 0.54 | -0.14 | <0.01 | 0.17 | -0.09 | 0.44 |

^a^Fisher’s Exact Test (two-sided)

^b^Spearman’s correlations (two-sided)

**Supplementary table 7a, b, c, d, e** Logistic regression of risk of having symptoms (total/work-related) for chimney sweeps (n=483) in relation to the extent of work (estimated as 10% increment of work fraction) in the past 12 months with 7a) inspection of fire-safety systems, 7b) cleaning ventilation channels, 7c) mandatory ventilation inspection, 7d) cleaning exhaust ducts and 7e) office work. Effects are presented as odds ratio (OR) in crude and adjusted models [95%CI= 95% confidence interval]

**Supplementary table 7a**

|  | Inspection of fire-safety systems | | | | | | | | | | | | | | | | |
| --- | --- | --- | --- | --- | --- | --- | --- | --- | --- | --- | --- | --- | --- | --- | --- | --- | --- |
| Symptoms | Total | | | | | | | | Work-related | | | | | | | |  |
|  | Crude | | | | Adjusted^a^ | | | | Crude | | | | Adjusted | | | |  |
|  | N | OR | 95%CI | | N | OR | 95%CI | | N | OR | 95%CI | | N | OR | 95%CI | |  |
| Wheeze^b^ | 449 | 1.01 | 0.92 | 1.11 | 138 | 1.02 | 0.87 | 1.20 | 63 | 0.94 | 0.82 | 1.08 | 26 | 0.94 | 0.74 | 1.19 |  |
| Cough^b^ | 453 | 0.96 | 0.91 | 1.02 | 138 | 0.94 | 0.85 | 1.05 | 136 | 1.02 | 0.89 | 1.16 | 43 | 0.99 | 0.81 | 1.21 |  |
| Nasal symptoms^bc^ | 452 | 1.02 | 0.96 | 1.07 | 137 | 0.98 | 0.88 | 1.08 | 197 | 0.97 | 0.89 | 1.05 | 63 | 0.93 | 0.80 | 1.07 |  |
| Eye symptoms^d^ | 450 | 0.99 | 0.93 | 1.05 | 449 | 0.99 | 0.93 | 1.10 | 124 | 0.85 | 0.75 | 0.96 | 124 | 0.85 | 0.75 | 0.96 |  |
| Nasal bleeding | 442 | 1.03 | 0.96 | 1.11 | 134 | 1.02 | 0.91 | 1.15 | 76 | 0.97 | 0.84 | 1.11 | 27 | 1.02 | 0.79 | 1.30 |  |

^a^Age, smoking status, and mask use (except for eye symptoms) were considered for adjustment.

^b^Without having a cold.

^c^Congestion, sneezing, itchy, or runny nose.

^d^Burning, stinging, runny, or itchy eye.

**Supplementary table 7b**

|  | Cleaning ventilation channel | | | | | | | | | | | | | | | | |
| --- | --- | --- | --- | --- | --- | --- | --- | --- | --- | --- | --- | --- | --- | --- | --- | --- | --- |
| Symptoms | Total | | | | | | | | Work-related | | | | | | | |  |
|  | Crude | | | | Adjusted | | | | Crude | | | | Adjusted | | | |  |
|  | N | OR | 95%CI | | N | OR | 95%CI | | N | OR | 95%CI | | N | OR | 95%CI | |  |
| Wheeze | 449 | 1.12 | 0.95 | 1.32 | 249 | 1.04 | 0.80 | 1.37 | 63 | 1.18 | 0.84 | 1.66 | 39 | 1.04 | 0.64 | 1.68 |  |
| Cough | 453 | 0.97 | 0.85 | 1.11 | 252 | 0.96 | 0.81 | 1.15 | 136 | 1.02 | 0.75 | 1.38 | 80 | 1.70 | 0.73 | 3.94 |  |
| Nasal symptoms | 452 | 1.04 | 0.92 | 1.17 | 251 | 1.09 | 0.93 | 1.28 | 197 | 0.99 | 0.84 | 1.18 | 117 | 1.06 | 0.83 | 1.35 |  |
| Eye symptoms | 450 | 1.03 | 0.90 | 1.17 | 449 | 1.04 | 0.91 | 1.18 | 124 | 1.27 | 0.98 | 1.65 | 124 | 1.28 | 0.99 | 1.67 |  |
| Nasal bleeding | 442 | 0.98 | 0.84 | 1.15 | 247 | 0.99 | 0.80 | 1.22 | 76 | 0.97 | 0.70 | 1.35 | 48 | 1.18 | 0.72 | 1.93 |  |

**Supplementary table 7c**

|  | Mandatory ventilation inspection* | | | | | | | | | | | | | | | | |
| --- | --- | --- | --- | --- | --- | --- | --- | --- | --- | --- | --- | --- | --- | --- | --- | --- | --- |
| Symptoms | Total | | | | | | | | Work-related | | | | | | | |  |
|  | Crude | | | | Adjusted | | | | Crude | | | | Adjusted | | | |  |
|  | N | OR | 95%CI | | N | OR | 95%CI | | N | OR | 95%CI | | N | OR | 95%CI | |  |
| Wheeze | 449 | 0.83 | 0.45 | 1.53 | 448 | 0.80 | 0.44 | 1.44 | 63 | 0.82 | 0.30 | 2.22 | 63 | 0.76 | 0.27 | 2.11 |  |
| Cough | 453 | 0.66 | 0.43 | 1.00 | 452 | 0.64 | 0.42 | 0.97 | 136 | 4.01 | 0.45 | 35.57 | 136 | 4.37 | 0.53 | 35.87 |  |
| Nasal symptoms | 452 | 1.10 | 0.83 | 1.46 | 451 | 1.11 | 0.84 | 1.48 | 197 | 0.86 | 0.58 | 1.28 | 197 | 0.85 | 0.58 | 1.26 |  |
| Eye symptoms | 450 | 1.16 | 0.87 | 1.55 | 449 | 1.17 | 0.87 | 1.57 | 124 | 1.04 | 0.68 | 1.58 | 124 | 1.02 | 0.67 | 1.56 |  |
| Nasal bleeding | 442 | 0.98 | 0.67 | 1.43 | 441 | 0.98 | 0.67 | 1.45 | 76 | 1.11 | 0.49 | 2.55 | 76 | 1.15 | 0.49 | 2.70 |  |

*The use of mask is not included in the adjustment because there was no variable for the use of mask in this task

**Supplementary table 7d**

|  | Cleaning exhaust ducts | | | | | | | | | | | | | | | | |
| --- | --- | --- | --- | --- | --- | --- | --- | --- | --- | --- | --- | --- | --- | --- | --- | --- | --- |
| Symptoms | Total | | | | | | | | Work-related | | | | | | | |  |
|  | Crude | | | | Adjusted | | | | Crude | | | | Adjusted | | | |  |
|  | N | OR | 95%CI | | N | OR | 95%CI | | N | OR | 95%CI | | N | OR | 95%CI | |  |
| Wheeze | 449 | 1.00 | 0.83 | 1.19 | 161 | 0.97 | 0.73 | 1.29 | 63 | 1.21 | 0.78 | 1.88 | 28 | 1.09 | 0.59 | 2.02 |  |
| Cough | 453 | 1.11 | 1.00 | 1.23 | 164 | 1.16 | 0.99 | 1.36 | 136 | 1.02 | 0.84 | 1.24 | 53 | 1.05 | 0.71 | 1.55 |  |
| Nasal symptoms | 452 | 0.96 | 0.86 | 1.07 | 163 | 0.89 | 0.76 | 1.04 | 197 | 1.05 | 0.88 | 1.26 | 78 | 1.26 | 0.90 | 1.76 |  |
| Eye symptoms | 450 | 1.01 | 0.90 | 1.14 | 449 | 0.99 | 0.89 | 1.12 | 124 | 1.25 | 0.99 | 1.58 | 124 | 1.25 | 0.99 | 1.57 |  |
| Nasal bleeding | 442 | 1.02 | 0.89 | 1.16 | 160 | 1.00 | 0.83 | 1.21 | 76 | 0.93 | 0.70 | 1.24 | 31 | 0.90 | 0.61 | 1.32 |  |

**Supplementary table 7e**

|  | Office work* | | | | | | | | | | | | | | | | |
| --- | --- | --- | --- | --- | --- | --- | --- | --- | --- | --- | --- | --- | --- | --- | --- | --- | --- |
| Symptoms | Total | | | | | | | | Work-related | | | | | | | |  |
|  | Crude | | | | Adjusted | | | | Crude | | | | Adjusted | | | |  |
|  | N | OR | 95%CI | | N | OR | 95%CI | | N | OR | 95%CI | | N | OR | 95%CI | |  |
| Wheeze | 449 | 0.91 | 0.75 | 1.11 | 448 | 0.89 | 0.73 | 1.09 | 63 | 0.77 | 0.55 | 1.09 | 63 | 0.74 | 0.52 | 1.05 |  |
| Cough | 453 | 0.92 | 0.83 | 1.03 | 452 | 0.93 | 0.84 | 1.03 | 136 | 0.85 | 0.69 | 1.03 | 136 | 0.87 | 0.71 | 1.06 |  |
| Nasal symptoms | 452 | 0.92 | 0.83 | 1.01 | 451 | 0.93 | 0.85 | 1.03 | 197 | 0.74 | 0.59 | 0.93 | 197 | 0.73 | 0.57 | 0.93 |  |
| Eye symptoms | 450 | 0.97 | 0.87 | 1.07 | 449 | 0.98 | 0.88 | 1.09 | 124 | 0.73 | 0.55 | 0.98 | 124 | 0.73 | 0.54 | 0.98 |  |
| Nasal bleeding | 442 | 1.02 | 0.92 | 1.14 | 441 | 1.04 | 0.94 | 1.16 | 76 | 0.84 | 0.66 | 1.06 | 76 | 0.84 | 0.66 | 1.08 |  |

*The use of mask is not included in the adjustment because there was no variable for the use of mask in this task
